# Case Report: Arthroscopic synovectomy and loose body removal for charcot knee in an adolescent with congenital insensitivity to pain with anhidrosis

**DOI:** 10.3389/fsurg.2026.1767073

**Published:** 2026-04-10

**Authors:** Qianjiang Xiong, Lian Huang, Yunan Hu, Weili Fu

**Affiliations:** 1 Sports Medicine Center, Department of Orthopedic Surgery/Orthopedic Research Institute, West China Hospital, Sichuan University, Chengdu, China; 2 West China School of Nursing, Sichuan University, Chengdu, Sichuan, China

**Keywords:** arthroscopic synovectomy, charcot knee, congenital insensitivity to pain with anhidrosis, joint preservation, loose body removal, neuropathic arthropathy, adolescent9

## Abstract

Charcot neuroarthropathy (CN) of the knee in adolescents with congenital insensitivity to pain with anhidrosis (CIPA) is exceptionally rare and presents unique diagnostic and therapeutic challenges. We present the case of a 13-year-old girl with genetically confirmed CIPA and a two-year history of recurrent knee swelling, effusion, and functional limitation. Imaging revealed intra-articular loose bodies and osteochondral defects in the left knee. The patient was treated with arthroscopic synovectomy and removal of a large loose body via standard anteromedial, anterolateral, and posteromedial portals. Intraoperative findings included extensive synovial proliferation and a 3 × 4 cm mobile loose body. Postoperatively, the patient followed a function-based rehabilitation protocol. At 30 days, she ambulated without assistive devices. At 1-year and 17-month follow-up, she demonstrated sustained clinical improvement with increased Lysholm score (from 50 to 82) and enhanced range of motion (from 10°–110° to 0°–140°). Magnetic resonance imaging (MRI) confirmed joint stability and absence of recurrent loose bodies. This case illustrates that arthroscopic management may offer a joint-preserving option in carefully selected adolescents with CIPA-associated Charcot knee and persistent mechanical symptoms. The approach may function as a temporizing measure to delay more invasive reconstructions. Adherence to protective joint education and close follow-up are essential in this population. This report highlights the clinical presentation, technical considerations, and mid-term outcomes of arthroscopic intervention in a rare neuropathic knee condition.

## Introduction

Charcot neuroarthropathy (CN) is a progressive, degenerative joint disorder characterized by osteoarticular destruction and instability, often leading to fractures and deformity (1). While it is most commonly documented in the setting of diabetic peripheral neuropathy and other acquired neuropathic conditions (2, 3), CN has also been reported in rare congenital sensory disorders, including Congenital Insensitivity to Pain with Anhidrosis (CIPA) (4–6). CIPA, also categorized as hereditary sensory and autonomic neuropathy (HSAN) type IV, is caused by neurotrophic receptor tyrosine kinase 1 (*NTRK1)* gene mutations that lead to absence of pain and temperature sensation and autonomic dysfunction (4). Classic clinical features include recurrent fractures, insensitivity to painful stimuli, and other musculoskeletal complications that arise from repetitive microtrauma (7–9).

Despite the well-recognized burden of Charcot arthropathy in adults, particularly in the foot and ankle (2, 3), the occurrence of Charcot changes in the knee is exceedingly uncommon, especially in adolescents with CIPA, with only sporadic case reports and small case series in the literature (10–15). Management strategies reported to date have varied widely, ranging from conservative care to joint-sacrificing procedures, and there is no consensus on optimal treatment for this rare pediatric presentation (13, 16–18). Furthermore, major reconstructive options such as total knee arthroplasty (TKA) and arthrodesis carry significant risks of complications and long-term functional limitations in young patients (16–19).

Arthroscopic synovectomy and removal of intra-articular loose bodies are well-established, minimally invasive techniques for addressing mechanical symptoms in other joint pathologies, but these approaches have been rarely described in the context of CIPA-associated Charcot knee (13, 14). In this report, we describe the clinical presentation, arthroscopic management, and mid-term functional outcome of a 13-year-old girl with genetically confirmed CIPA and symptomatic Charcot knee. This case illustrates technical considerations, clinical decision-making, and potential value of a joint-preserving strategy in a condition where evidence is limited.

## Case description

A 13-year-old girl presented to our tertiary referral center with a two-year history of recurrent left knee swelling that had progressively worsened over the three weeks prior to evaluation. Her guardians reported intermittent knee effusion and a gradually declining range of motion (ROM), defined here as the active range measured clinically, without a clear recent traumatic trigger. Her past medical history was notable for CIPA, confirmed previously by whole-exome sequencing demonstrating compound heterozygous mutations in the *NTRK1* gene. There was no known family history of similar sensory neuropathies. Early manifestations of her underlying condition included multiple fractures beginning at two months of age and terminal finger stumps since birth (Supplementary Figure S1), consistent with her profound sensory deficit. At age 12, she underwent open reduction and internal fixation for a right femoral shaft fracture. The left knee had initially been managed non-operatively with intermittent aspirations after an osteochondral defect was diagnosed following a hiking injury at approximately 11 years of age.

**Figure 1:**
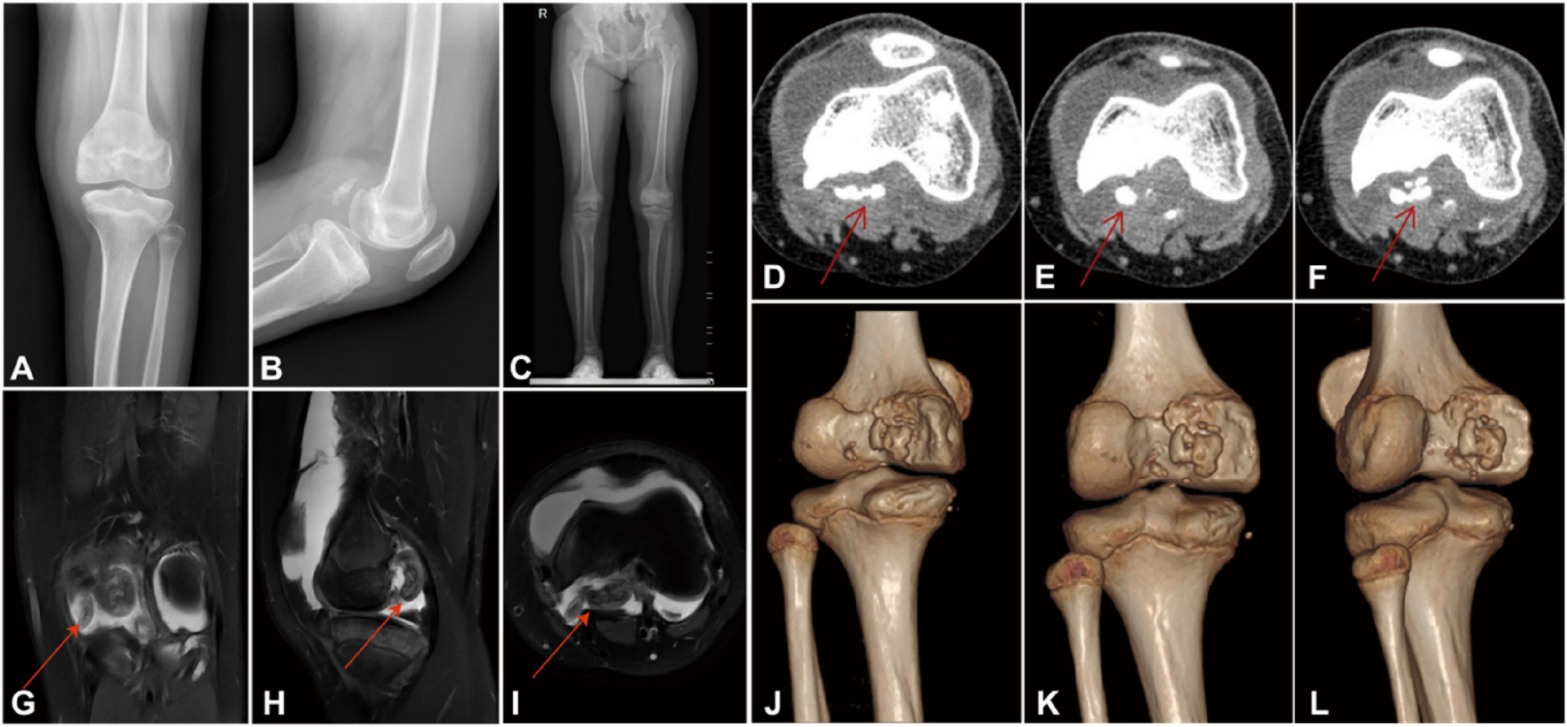
Preoperative imaging of the left knee in a 13-year-old girl with CIPA-associated charcot neuroarthropathy. **(A–C)** Anteroposterior **(A)**, lateral **(B)**, and full-length standing **(C)** radiographs demonstrate joint effusion, soft-tissue swelling, and irregular articular surfaces; the long-leg view provides overall coronal limb alignment. **(D–F)** Sequential axial CT (bone window) slices show multiple ossified intra-articular loose bodies in the posterior compartment (red arrows) with subchondral/cortical irregularities, better defining the osseous fragmentation. **(G–I)** Knee MRI on fluid-sensitive (PD/T2-fat-suppressed) sequences reveals massive joint effusion, synovial thickening, and extensive bone-marrow edema. Multiple low-signal intra-articular loose bodies are identified in the posterior compartment (red arrows) on coronal **(G)**, sagittal **(H)**, and axial **(I)** planes. **(J–L)** 3D CT reconstructions provide comprehensive visualization of osseous fragmentation/loose bodies and articular surface destruction, complementing the axial CT findings.

On clinical examination, neurological evaluation revealed a marked loss of pain and temperature sensation, evidenced by lack of avoidance or withdrawal responses to noxious pinprick and heat stimulation. Clinically tested vibration sense (128 Hz tuning fork) and proprioception (joint position sense) were relatively preserved, consistent with CIPA phenotypes in which large-fiber modalities remain intact. Deep tendon reflexes (patellar and Achilles) were present but mildly diminished. Autonomic evaluation confirmed anhidrosis and labile temperature responses without other focal autonomic deficits on examination. Locally, the left knee was markedly swollen with a positive floating patella sign and restricted motion, with flexion limited to 110° and an extension deficit of 10°. Ligamentous stability tests, including anterior drawer and Lachman tests, were unremarkable. Laboratory workup revealed no significant abnormalities, with normal fasting blood glucose (4.45 mmol/L) and calcium (2.25 mmol/L). Systemic inflammatory markers and blood counts were within normal limits or only mildly elevated, including a white blood cell count of 8.91 × 10^9^/L, a platelet count of 273 × 10^9^/L, a C-reactive protein level of 2.86 mg/L, and an erythrocyte sedimentation rate of 22 mm/h. Comprehensive serological screening was negative. These unremarkable laboratory findings, alongside the absence of constitutional symptoms, made active infectious or systemic autoimmune etiologies such as septic arthritis and juvenile idiopathic arthritis (JIA) unlikely. Furthermore, because the aspirated synovial fluid was clear and the underlying neuropathic etiology was definitively established via genetic testing (whole-exome sequencing), intraoperative cultures were not obtained given the low clinical suspicion for infection. While osteochondritis dissecans (OCD) was considered in the differential diagnosis due to her age and the presence of loose bodies, the extensive neuropathic joint destruction and profound sensory deficit were more consistent with CIPA-associated Charcot arthropathy. Plain radiographs of the affected knee demonstrated an osteochondral defect of the medial femoral condyle and irregularities of the proximal tibial cortex. Computed tomography confirmed cortical irregularities and significant effusion, while magnetic resonance imaging (MRI) revealed multiple intra-articular loose bodies and extensive joint fluid. These findings were consistent with an active destructive neuropathic arthropathy process entering Eichenholtz Stage II (coalescence), characterized by fragmented bone with blunt margins and loose body formation without mature consolidation (Figure 1). Radiographs of the contralateral femur showed stable internal fixation consistent with her prior surgical history (Supplementary Figure S2).

**Figure 2:**
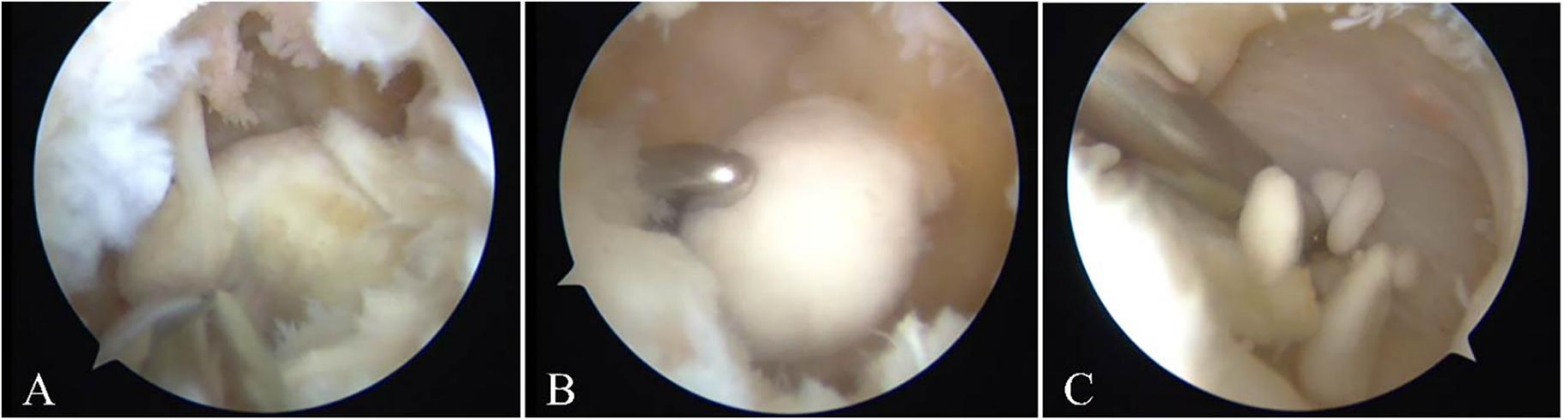
Intraoperative arthroscopic views of the left knee: **(A)** hypertrophic and inflamed synovium; **(B)** a large, ossified loose body is identified in the posterior compartment; **(C)** the loose body is grasped with forceps for removal.

Given her persistent mechanical symptoms and functional impairment, arthroscopic synovectomy and loose body removal were performed in April 2024 under standard perioperative antibiotic prophylaxis. Intraoperatively, extensive hyperplastic synovial tissue and severe degeneration of the medial meniscus body were encountered. The synovium was meticulously debrided, and a medial meniscus plasty was performed to excise the irreparable tissue via standard anteromedial and anterolateral portals. A posteromedial trans-septal portal was established to access the posterior compartment, where a large, mobile loose body measuring approximately 3 × 4 cm was identified and successfully extracted in its entirety (Figure 2). The articular surfaces were inspected, revealing localized cartilage wear on the medial femoral condyle. Unstable cartilage flaps and intra-articular debris were debrided with a paring instrument. No arthrotomy was required, and the underlying joint architecture was preserved. Postoperative radiographs confirmed complete removal of the loose body (Supplementary Figure S3), and pathological examination of the removed tissue did not demonstrate specific histopathological abnormalities.

Postoperatively, the diagnosis of CIPA necessitated a strictly time- and function-based rehabilitation program, as her lack of protective pain sensation precluded symptom-guided progression. Although there is no universally standardized knee arthroscopy rehabilitation protocol, especially in the context of neuropathic sensory loss, we established a time- and function-based progression tailored to this patient, with objective ROM milestones and bracing guidelines. A standard hinged knee brace was employed as the primary orthotic device. No custom orthoses or specialized footwear were prescribed. The brace was mandated for all weight-bearing ambulation during the first 2–3 weeks to mechanically shield the joint from unperceived abnormal loading. The patient demonstrated excellent adherence to this bracing protocol with no reported non-compliance. To prevent unperceived pressure injuries and subsequent soft-tissue infections, rigorous daily skin inspections were performed by caregivers under the brace and the two-week elastic compression bandage.

Early rehabilitation commenced immediately with quadriceps isometric contractions, straight leg raises, and ankle pumps. For this specific case, we set safety-oriented ROM targets of 0°–90° for the first two weeks, progressing to 0°–120° by four weeks postoperatively. At the two-week follow-up, a repeat joint aspiration was performed, yielding approximately 70 mL of clear synovial fluid. There were no clinical or laboratory signs of infection at that time, the fluid remained clear, and inflammatory markers were not elevated, consistent with non-infectious postoperative effusion rather than septic arthritis. No additional therapeutic aspirations were required thereafter. Based strictly on objective clinical milestones rather than subjective feedback, she progressed to partial weight-bearing. By 30 days postoperatively, she ambulated without assistive devices. At the 1-year follow-up, her Lysholm knee score demonstrated a clinically meaningful improvement from 50 to 82, and her ROM reached 0°–140°. At the 17-month follow-up, she remained free of mechanical symptoms or recurrent effusion, with follow-up MRI confirming a sustained absence of loose bodies and stable articular contours, indicating a successful transition to a stable Eichenholtz Stage III (consolidation) without the need for radical surgery (Supplementary Figure S4). Crucially, high-impact activities and contact sports were strictly restricted. Throughout follow-up, lifelong patient and family education was emphasized regarding joint protection and the avoidance of high-stress postures (e.g., prolonged cross-legged sitting) to mitigate recurrent microtrauma.

**Figure 3:**
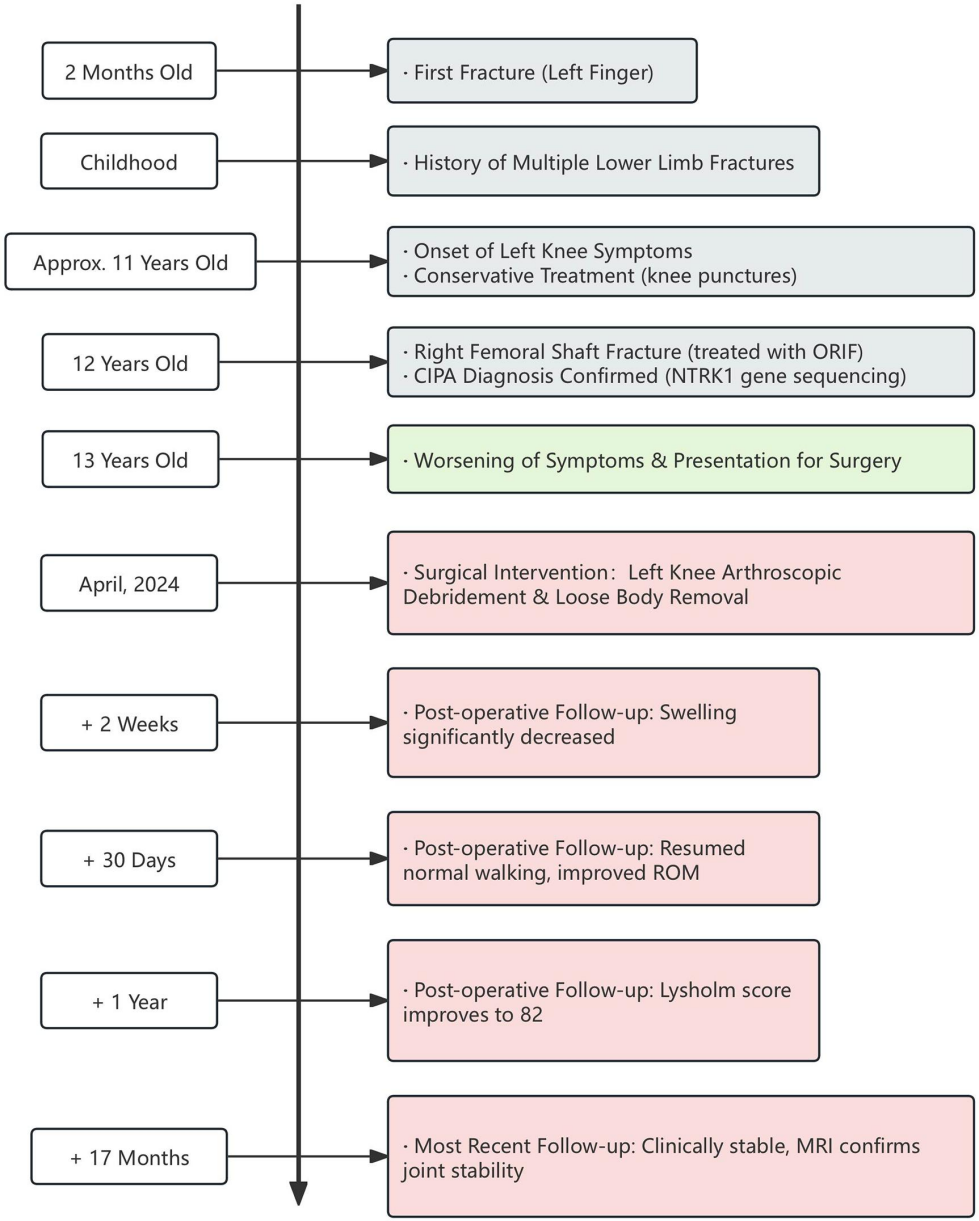
Timeline of key clinical events. The timeline illustrates the patient's complete medical history, from her first fracture at 2 months of age through the key diagnostic and surgical events, to the final 17-month postoperative follow-up, which includes both functional and imaging outcomes. (CIPA, congenital insensitivity to pain with anhidrosis; ORIF, open reduction and internal fixation; ROM, range of motion; MRI, magnetic resonance imaging).

No intraoperative or postoperative complications were observed, and no adverse events were reported during the 17-month follow-up period. The patient's functional gains were maintained, enabling meaningful participation in age-appropriate activities and daily life as confirmed by both objective measures and guardian reports (Supplementary Table S1). A comprehensive timeline of the episode of care is presented in Figure 3.

## Discussion

CN is a destructive joint condition that arises from sensory and autonomic dysfunction, resulting in progressive arthropathy, joint instability, and deformity (1). When CN occurs in the setting of CIPA, the clinical challenge is amplified by profound sensory deficits and a lifelong predisposition to repetitive microtrauma. CIPA is caused by loss-of-function mutations in the *NTRK1* gene, which impair nociceptive and autonomic pathways and lead to a high burden of skeletal complications (4, 5, 10, 11). Despite this known propensity for skeletal injury, specific reports of Charcot knee in adolescents with CIPA are rare, and there is no established consensus on optimal management strategies (7, 8, 10–15).

The current literature on CIPA-associated Charcot arthropathy largely consists of isolated case reports and small case series (7, 8, 10–15). A summary of previously reported cases specifically involving the knee joint is provided in Supplementary Table S2 (6, 13, 14, 20). Several of these describe neuropathic joint involvement with varied anatomical distribution, including foot, ankle, and knee joints. Notably, Alghamdi et al. reported arthroplasty in a Charcot knee in a pediatric patient, highlighting the complexity and high morbidity of joint-sacrificing approaches in this population (13). Other case reports emphasize conservative measures or combinations of bracing and limited surgical intervention but do not extensively address minimally invasive, joint-preserving techniques (8, 14, 15, 21). These fragmented clinical experiences underscore the paucity of evidence and the need for individualized decision-making in adolescents with CIPA and destructive joint pathology.

TKA and arthrodesis have been used as definitive surgical strategies in severe Charcot knees. However, these approaches are associated with significant complication rates and functional limitations, particularly in young patients with long remaining life expectancy (12, 13, 16, 21, 22). For example, Rattanaprichavej et al. reported that contemporary TKAs in Charcot patients carry elevated risks of instability, infection, periprosthetic fracture, and loosening (16). Likewise, older reports have described arthrodesis as a salvage option for neuropathic joints that may be associated with complications and variable functional outcomes, reflecting the trade-offs between mechanical stability and long-term mobility (12, 22, 23). Given these limitations, less invasive, joint-preserving options warrant investigation to address mechanical symptoms without prematurely committing to irreversible reconstruction in skeletally immature individuals.

Accurate clinical staging is crucial for guiding surgical interventions in CN. The Eichenholtz classification is a widely accepted temporal staging system that describes the natural progression of Charcot arthropathy based on radiographic and clinical evolution, and it can assist clinicians in diagnosis, staging, and treatment planning across affected joints, including the knee (24). At the time of surgery, our patient's presentation was best described as entering Eichenholtz Stage II (coalescence). Given that the process remained subacute with ongoing bone turnover and incomplete fragment fusion, yet preserved overall joint alignment, a joint-preserving approach was preferable. The presence of extensive bone-marrow edema indicated an active or subacute inflammatory phase. Although surgical intervention is traditionally approached with caution in this setting, this risk was carefully weighed against the severe ongoing damage caused by the massive intra-articular loose body. We reasoned that the loose body was acting as a relentless mechanical irritant, perpetuating the inflammatory cycle, as large intra-articular loose bodies and synovial proliferation can themselves perpetuate the cycle of effusion, local inflammation, and structural deterioration (25). By utilizing a minimally invasive arthroscopic technique rather than an open arthrotomy, we mitigated the surgical trauma while effectively removing both the mechanical (loose body) and biological (hyperplastic synovium) drivers of joint destruction. More radical reconstructions, such as arthrodesis or total joint replacement, are typically reserved for Stage III consolidated joints with fixed deformity and structural instability, as these procedures require a stable structural platform and carry higher complication rates, particularly in younger patients where implant survivorship and mechanical demands pose additional concerns (26, 27).

In this context, arthroscopic synovectomy and loose body removal provide a minimally invasive, joint-preserving strategy. Notably, intra-articular biological adjuncts (e.g., corticosteroids, platelet-rich plasma) were deliberately avoided, as their efficacy is primarily documented in degenerative osteoarthritis and remains unproven in pediatric Charcot neuro-osteoarthropathy (28). Furthermore, concurrent cartilage restoration procedures (e.g., microfracture or osteochondral grafting) were deliberately deferred. In the pediatric knee, treatment decisions must carefully weigh fragment stability, defect characteristics, and skeletal maturity. Because the massive osteochondral loose body was chronic and irreducible, primary fixation was precluded. While cartilage repair techniques are viable for focal, well-defined lesions in adolescents, they are generally ill-suited for extensive, longstanding neuropathic fragmentation (29, 30). Moreover, techniques requiring subchondral bone penetration may raise theoretical concerns in skeletally immature patients regarding potential physeal disruption and altered articular development, though this has not been definitively established in pediatric neuropathic arthropathy contexts (31). Thus, isolated excision was prioritized to immediately relieve the mechanical block and mitigate secondary cartilage wear without inflicting potential iatrogenic trauma.

By targeting the main mechanical sources of disability, including recurrent effusion, problematic loose bodies, and synovial proliferation, this arthroscopic technique aims to preserve the native joint architecture and mitigate progressive structural deterioration. In the present case, this approach was associated with substantial and sustained clinical improvement, as reflected by a clinically meaningful increase in Lysholm knee score and enhanced ROM at both 1-year and 17-month follow-up. These findings complement the limited reports of arthroscopic management in neuropathic knees and suggest that, in select adolescents with CIPA and preserved alignment without gross subluxation or severe collapse, arthroscopy can provide durable symptom relief without the morbidity associated with open, joint-sacrificing procedures (13, 14). In this case, we performed arthroscopic synovectomy and loose body removal in an adolescent with CIPA-associated Charcot knee, achieving sustained symptomatic relief and significant functional improvement at both 1-year and 17-month follow-up. These outcomes suggest that in selected patients with preserved alignment and mechanical symptoms, a minimally invasive, joint-preserving strategy may be a valuable therapeutic option, potentially delaying the need for joint-sacrificing reconstructions.

### Management framework and decision guide

**Figure 4:**
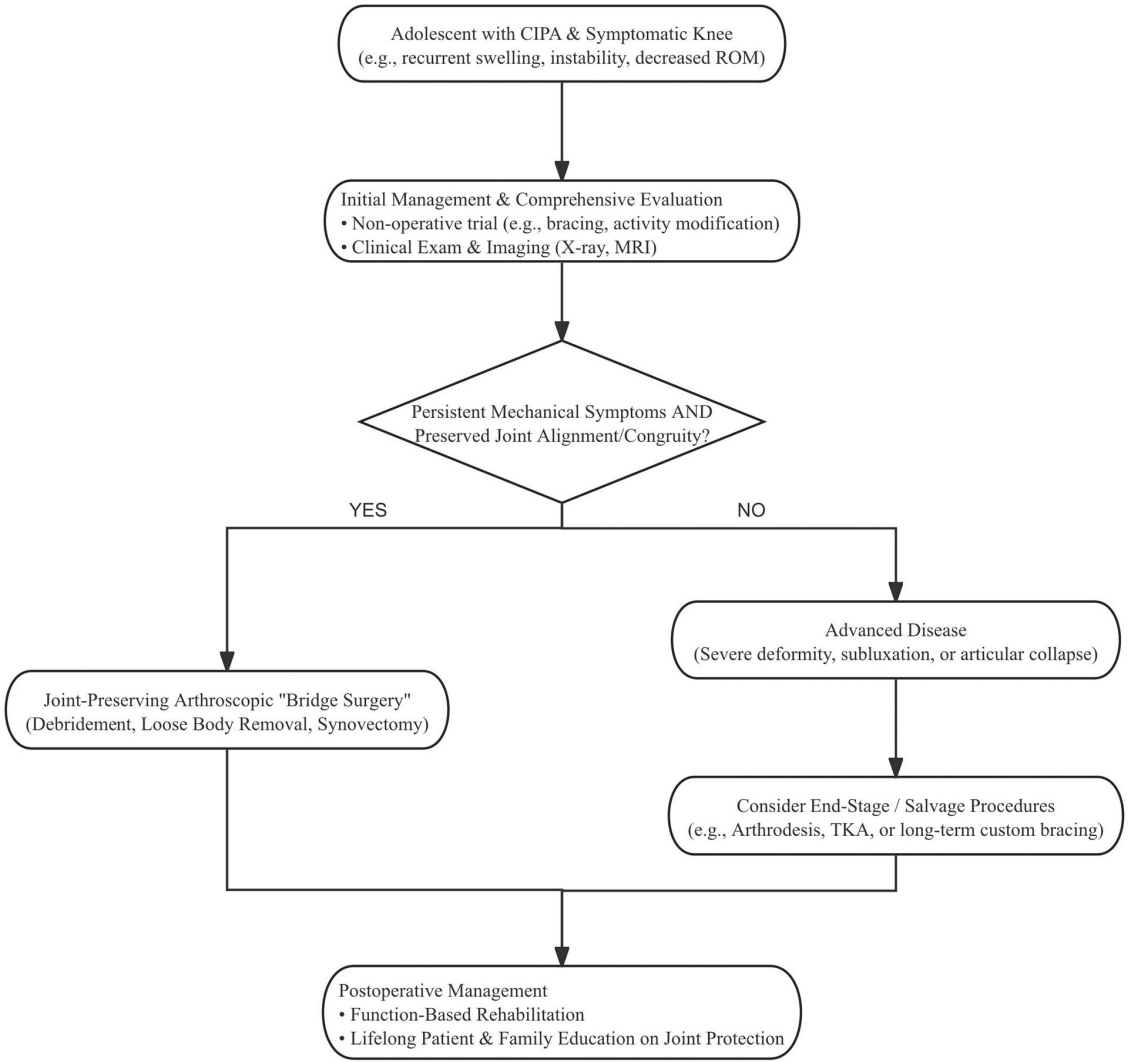
A proposed management framework for CIPA-associated charcot knee in adolescents. The framework emphasizes a joint-preserving “bridge surgery” for patients with mechanical symptoms and preserved joint alignment, while reserving end-stage procedures for those with advanced deformity. Lifelong patient education remains the cornerstone of management regardless of the treatment path. (CIPA, congenital insensitivity to pain with anhidrosis; MRI, magnetic resonance imaging; ROM, range of motion; TKA, total knee arthroplasty.

To guide clinical decision-making in this rare and complex scenario, we propose a management framework and decision guide synthesized from the literature and our experience (Figure 4). This framework suggests that for adolescents presenting with persistent mechanical symptoms but preserved joint alignment, arthroscopic intervention should be utilized as a temporizing measure. This strategy aims to ameliorate mechanical pathology and enhance quality of life, effectively deferring more aggressive reconstructions until skeletal maturity or structural collapse dictates otherwise.

Furthermore, it is important to clarify the intended scope of the proposed management framework. Although developed in the specific context of Charcot knee associated with CIPA, the fundamental orthopedic principles underlying this algorithm are conceptually relevant to other neuropathic arthropathies, such as prioritizing joint-preserving interventions for persistent mechanical symptoms in the setting of preserved alignment and deferring joint-sacrificing reconstruction in skeletally immature patients. Neuropathic arthropathy broadly refers to joint destruction arising from peripheral neuropathy and repetitive microtrauma due to loss of protective sensation and can be caused by diverse etiologies including diabetic neuropathy, myelomeningocele, leprosy, and HSAN variants. Although current international evidence-based guidelines for diabetic Charcot neuro-osteoarthropathy emphasize individualized off-loading, monitoring, and surgical timing based on disease activity and comorbidity, these recommendations cannot be directly extrapolated but must be conceptually adapted to CIPA-associated neuropathic arthropathy, with postoperative care tailored to its distinct sensory and autonomic profile (28). However, the specific recommendations for postoperative care must be tailored to the underlying sensory and autonomic profile. For example, while intensive daily skin inspection and protective bracing are critical in CIPA due to anhidrosis and absent pain, diabetic patients may retain some autonomic function but face distinct wound-healing, vascular, and metabolic challenges that influence off-loading strategies and orthotic needs. Consequently, clinicians applying this framework to non-CIPA neuropathies should adapt postoperative management to the specific pathophysiology and clinical context of the underlying disease (28).

### Postoperative precautions and rehabilitation strategy

Importantly, the diagnosis of CIPA must significantly influence postoperative precautions. CIPA is characterized by an inability to perceive pain and temperature and by anhidrosis, which predisposes patients to unrecognized injuries, repetitive microtrauma, and delayed recognition of complications. This underlying pathophysiology necessitates critical modifications to standard postoperative protocols because rehabilitation progression cannot be guided by symptom tolerance or pain feedback alone. In general knee arthroscopy practice, rehabilitation is typically phase-based and criterion-based, with progression of weight-bearing, range of motion, and muscle strengthening guided by objective functional milestones rather than pain alone. Most providers permit early ROM from 0° to 90°immediately after arthroscopy and advance full ROM over several weeks, and bracing and weight-bearing recommendations are adjusted according to surgical context and healing response (32, 33). Early incorporation of quadriceps activation, motion exercises and controlled progression is widely recommended to restore strength and prevent stiffness in arthroscopic recovery protocols (34, 35). Given the patient's young age and early mobilization, chemical thromboprophylaxis was not administered. Instead, mechanical prophylaxis via early ankle pumps was utilized.

As aligned with our management framework (Figure 4), we advocate for a strictly time- and function-based rehabilitation program rather than a symptom-guided one. Furthermore, sensory-specific precautions are fundamental adaptations to the CIPA phenotype. These include mandated protective bracing to limit unperceived abnormal loading, and rigorous daily skin inspections to prevent pressure injuries associated with anhidrosis. Finally, lifelong education on joint protection and the avoidance of high-stress activities (e.g., sitting cross-legged) is essential to reduce recurrent microtrauma and prolong the functional lifespan of the native joint.

### Patient-reported outcomes and quality of life

Beyond objective functional measures, capturing the patient's lived experience is essential for understanding the full impact of interventions in neuropathic conditions. Although pain scores are not applicable in CIPA, disturbances in daily activities, swelling recurrence, and participation limitations are meaningful to patients and families. During follow-up, emphasis was placed on patient and family education regarding joint protection strategies. The patient's legal guardians described the real-world impact of the combined surgical and educational approach, stating: “For the first time in many years, we don't have to worry about her knee swelling up after just a short walk. She can now participate in school activities with her friends—something we thought would never be possible.”

### Limitations and future directions

This case report has inherent limitations that must be acknowledged. First, as a single patient experience, the findings may not be generalizable to all adolescents with CIPA or to other neuropathic etiologies of Charcot arthropathy. Second, although the 17-month follow-up MRI (Supplementary Figure S4) showed stable joint space without clinically silent subluxation or obvious structural collapse, we lack comparative pre- and post-operative full-length weight-bearing radiographs to precisely quantify longitudinal alignment changes (e.g., specific varus/valgus angles or mechanical axis deviation). Consequently, proposing strict numerical cutoffs for joint-preserving surgery based on this single case would be premature. Third, our functional assessment relied on the Lysholm score and ROM. The absence of validated pediatric or neuropathy-specific outcome measures [e.g., the Childhood Health Assessment Questionnaire (CHAQ), the Pediatric Quality of Life Inventory (PedsQL)] limits the comprehensiveness of our evaluation and should be addressed in future studies. Fourth, longer follow-up is required to assess the ultimate durability of this arthroscopic intervention and monitor for late structural progression. Finally, while arthroscopy carries a theoretical risk of accelerating degeneration in neuropathic joints, this was carefully weighed against the immediate necessity of alleviating severe mechanical impingement.

Regarding future directions, the integration of wearable sensors or activity monitors represents a highly impactful area of ongoing research. Because CIPA patients cannot rely on pain as a feedback mechanism, developing mature wearable technologies to objectively track joint loading and alert caregivers to excessive stress could revolutionize the conservative and postoperative management of this exceptionally challenging condition. Finally, while the proposed algorithm (Figure 4) is informed by current evidence, prospective validation in larger cohorts is necessary to refine clinical decision thresholds.

## Conclusion

In conclusion, this case demonstrates that arthroscopic synovectomy and loose body removal can achieve sustained symptomatic relief and functional improvement in an adolescent with CIPA-associated Charcot knee, with preserved joint alignment and without the morbidity of open, joint-sacrificing procedures. The favorable 17-month follow-up suggests that in selected patients, this minimally invasive, joint-preserving strategy may serve as an effective temporizing option, potentially delaying or avoiding radical reconstruction until skeletal maturity or structural collapse necessitates it. The proposed management framework and decision guide offers clinicians a structured approach to stratifying mechanical symptoms and long-term joint preservation in this rare and complex clinical context. Further studies with larger cohorts and extended follow-up are needed to confirm these findings and refine criteria for selecting joint-preserving approaches in this rare and challenging condition.

## Data Availability

The original contributions presented in the study are included in the article/Supplementary Material, further inquiries can be directed to the corresponding author.
