# Supplementary material for: Case Report: Arthroscopic synovectomy and loose body removal for charcot knee in an adolescent with congenital insensitivity to pain with anhidrosis
Source: Front Surg. 2026 Apr 10;13:1767073. doi: 10.3389/fsurg.2026.1767073 (PMC13106444; doi:10.3389/fsurg.2026.1767073)
Supplement: Supplementary file 1 [file Supplementaryfile1.pdf]

Supplementary Table S1 *Summary of clinical and functional outcomes*

Follow-up Time Point	Lysholm Score	Range of Motion (°)	Clinical Observations
Preoperative	50	10–110	Marked swelling
2 Weeks Postoperative	65	0–115	Mild swelling
30 Days Postoperative	75	0–120	No pain, mild swelling
1 Year Postoperative	82	0–140	No mechanical symptoms, no significant swelling
17 Months Postoperative	-	0–140	Sustained clinical stability; MRI confirmed no recurrent loose bodies

Supplementary Table S2 *Published reports of knee and peri-knee manifestations in CIPA/HSAN-IV (including Charcot knee)*

Study	Age	Sex	Diagnostic Basis	Knee Involvement / Pathology	Intervention for the Knee	Key take-home relevant to this report
Abell (1964) (6)	NR	NR	Clinical diagnosis of congenital insensitivity to pain	Neuropathic degenerative arthritis of the knee (Charcot knee)	Arthrodesis	Foundational report establishing that Charcot knee occurs in CIPA; sets a historical precedent for salvage surgery when joint destruction is advanced.
Alghamdi (2022) (13)	13	Female	Genetic testing (NTRK1 mutation)	Recurrent swelling and instability without pain (diagnosed as Charcot knee)	Hinged total knee arthroplasty	Illustrates the feasibility of joint-sacrificing reconstruction in pediatric CIPA, but simultaneously highlights why joint-preserving options are highly attractive to delay such radical procedures in youth.
Batouk (2021) (20)	15	Male	Clinical CIPA phenotype	Multiple destructed joints including both knees (multi-joint Charcot pattern)	Conservative/supportive management (walking aids reported)	Demonstrates progressive joint destruction and a high functional burden despite non-operative care; supports the need for individualized, joint-specific, and timely surgical decision-making.
Kohler (2023) (14)	10	Female	Clinical diagnosis (HSAN-IV)	Orthopedic manifestations include proximal patellar sleeve fracture; highlights complexity of peri-/knee region injuries in HSAN-IV	Not knee-specific Charcot surgery reported; emphasizes management challenges and complications	Highlights management difficulties, the importance of early diagnosis, and the need for caution regarding postoperative restriction adherence; supports tailoring postoperative protocols (bracing/skin checks) to the specific neuropathy phenotype.

Note: NR = Not Reported / Not Retrieved in scoping context; CIPA = Congenital Insensitivity to Pain with Anhidrosis; HSAN-IV = Hereditary Sensory and Autonomic Neuropathy type IV

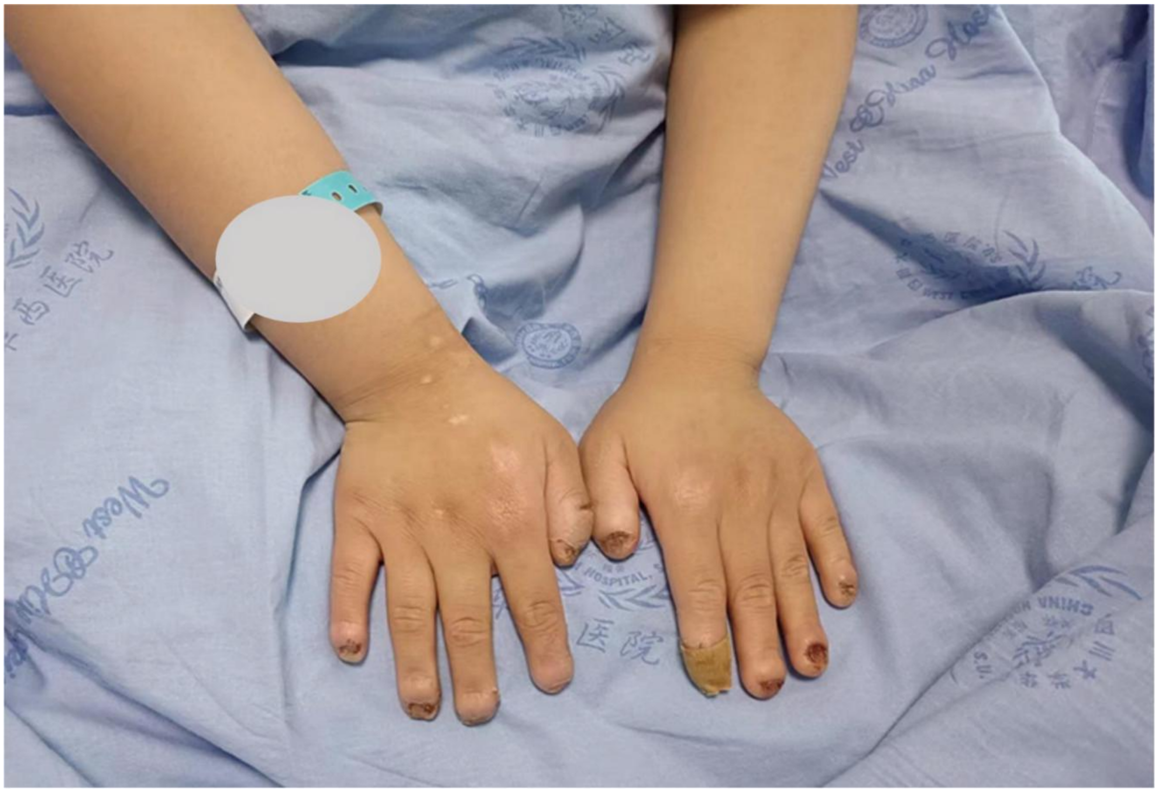

Supplementary Figure S1. Clinical photograph demonstrating the characteristic blunted fingertips (acromutilation) in the patient with CIPA.

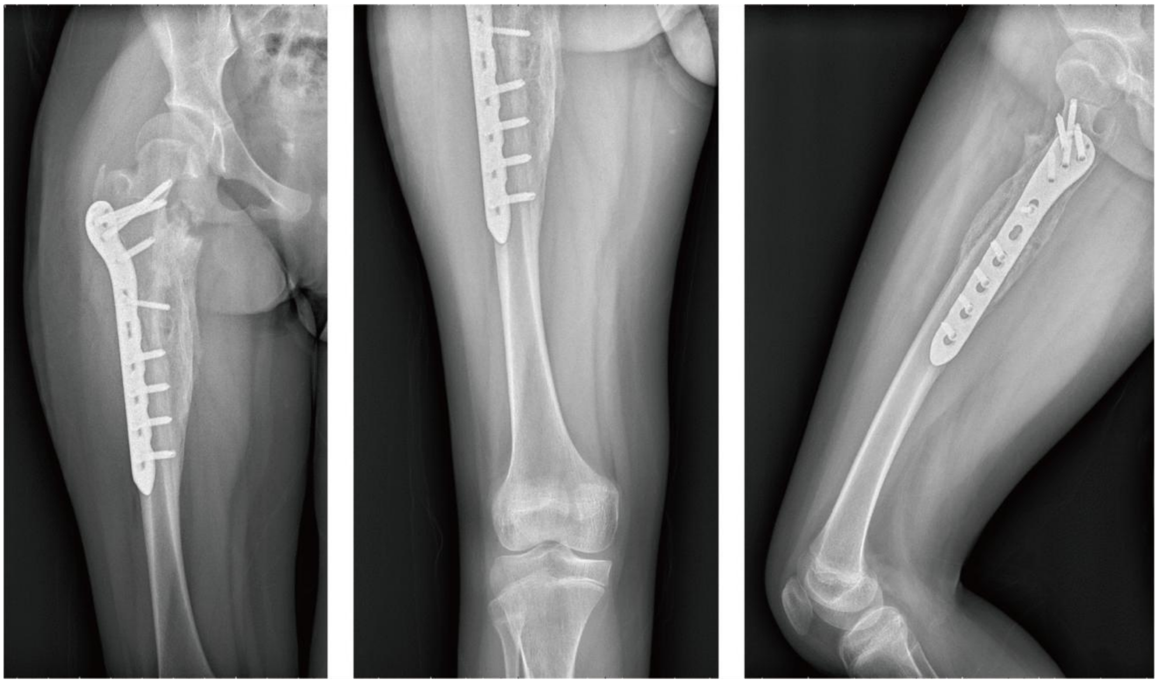

Supplementary Figure S2. Radiographs of the contralateral (right) femur, showing the status of internal fixation from a prior femoral shaft fracture surgery.

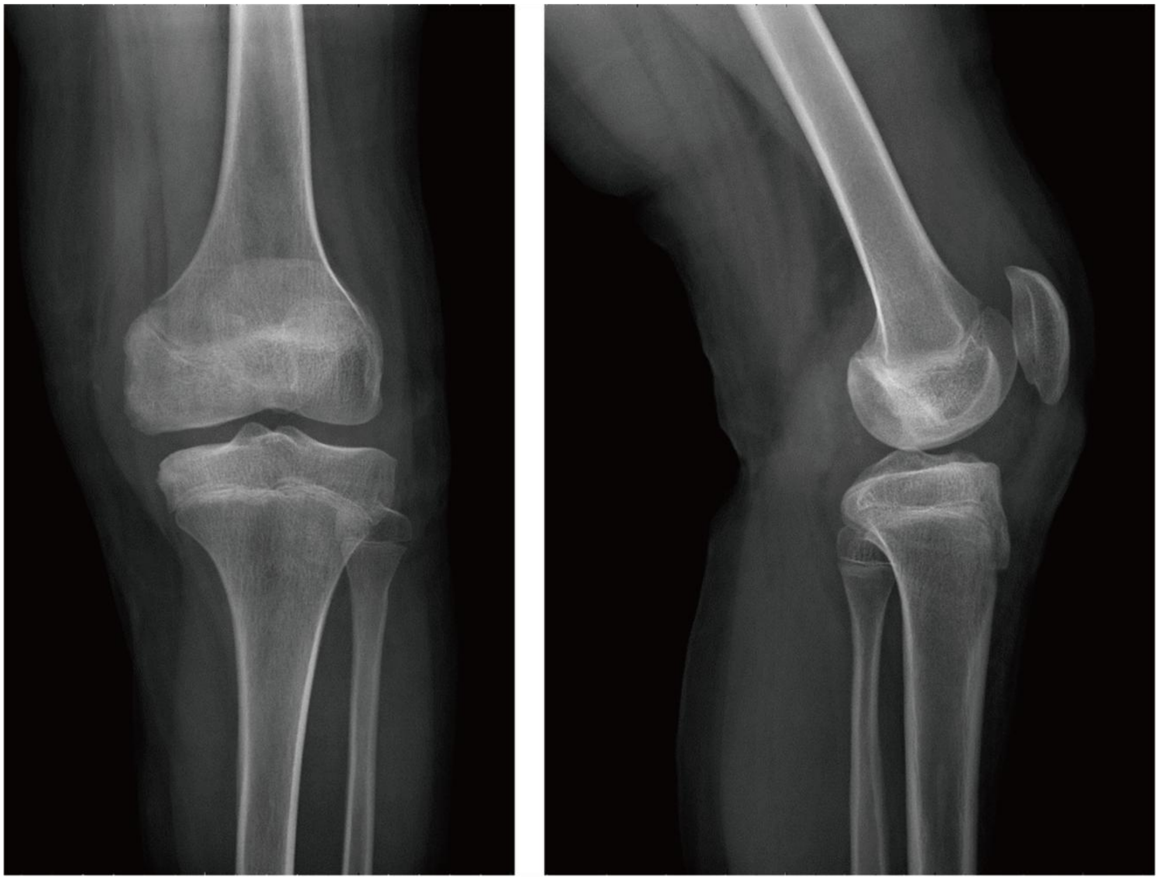

Supplementary Figure S3. Immediate postoperative anteroposterior (left) and lateral (right) radiographs of the left knee, confirming the complete removal of the large intra-articular loose bodies.

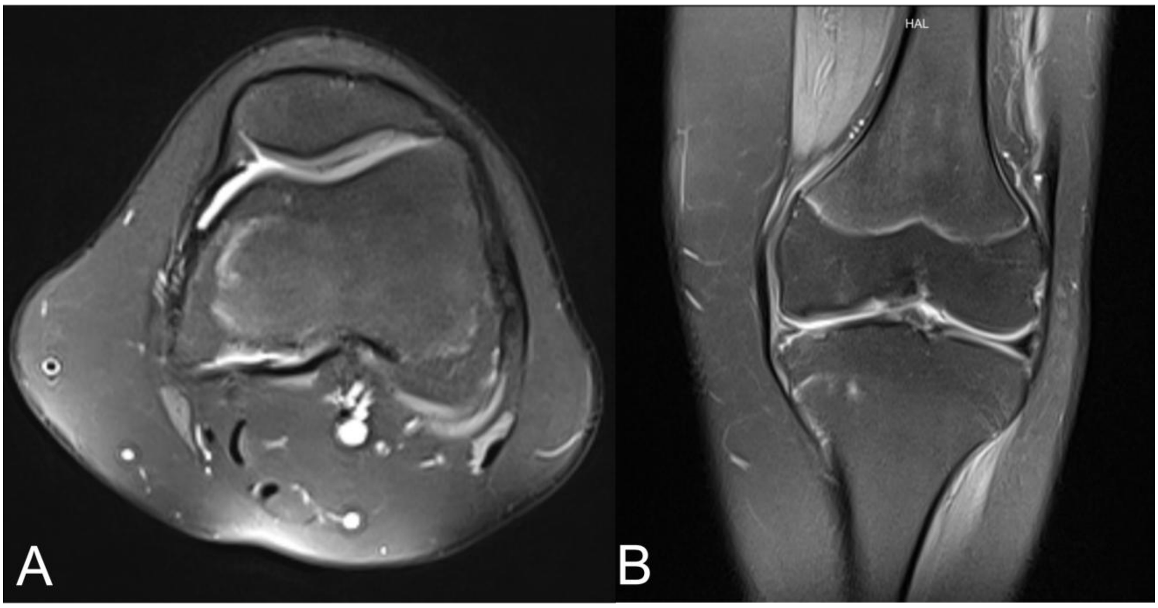

Supplementary Figure S4. Magnetic resonance imaging (MRI) of the left knee at 17 months postoperatively. (A) Axial and (B) coronal fat-suppressed proton density-weighted images demonstrate the complete absence of any recurrent large intra-articular loose bodies. Compared to preoperative imaging, there is a significant reduction in joint effusion and synovial inflammation. Importantly, the articular cartilage surfaces remain stable without evidence of progressive full-thickness cartilage loss or subchondral collapse, confirming mid-term joint stability.
